# Glaucoma after penetrating keratoplasty

**DOI:** 10.22336/rjo.2017.30

**Published:** 2017

**Authors:** Mihail Zemba, Alina-Cristina Stamate

**Affiliations:** * Department of Ophthalmology, “Dr. Carol Davila” Central Military Emergency University Hospital, Bucharest, Romania; ** “Carol Davila” University of Medicine and Pharmacy, Bucharest, Romania; *** Arena Med Clinic, Bucharest, Romania

**Keywords:** penetrating keratoplasty, high intraocular pressure, glaucoma post penetrating keratoplasty, antiglaucomatous therapy

## Abstract

Penetrating keratoplasty is a surgical intervention that despite the progress of surgical techniques and of postoperative treatment continues to have numerous complications. Many of them, such as graft rejection, significant astigmatism, cystoid macular edema, or cataract lead to important limitations of the visual function. Glaucoma is possibly the most dangerous complication following PK, leading to loss of the visual potential of the eye due to irreversible damage to the optic nerve. Identifying the risk factors permits an attentive follow-up and rapid treatment of the postoperative IOP rises. Maybe the most important is that preexisting glaucoma should be rightly diagnosed and controlled before PK, medically or, if necessary, surgically.

**Abbreviations:** PK = penetrating keratoplasty, IOP = intraocular pressure, PAS = peripheral anterior synechiae, TM = trabecular meshwork, DM = Descemet membrane, GAT = Goldmann applanation tonometry, MMC = mitomycin C, CAI = carbonic anhydrase inhibitors, 5-FU = 5-fluorouracil

## Introduction

Penetrating keratoplasty (PK) is a surgical intervention that despite the progress of surgical techniques and of postoperative treatment continues to have numerous complications. Many of them, such as graft rejection, significant astigmatism, cystoid macular edema, or cataract lead to important limitations of the visual function. Glaucoma is possibly the most dangerous complication following PK, leading to loss of the visual potential of the eye due to irreversible damage to the optic nerve, contrary to other complications, in which visual recovery can be expected.

Glaucoma following PK has a relatively high frequency, it can appear early, as well as late in the evolution of the transplant, it is very hard to diagnose and also to follow-up and the medical or surgical treatment can interfere negatively with the evolution of the corneal graft [**1**].

## Incidence

In 1969, Irvine and Kaufman were the first to publish a study that emphasized the increased incidence of high intraocular pressure (IOP) after PK. The maximum mean IOP in the first postoperative week was 24 mmHg in phakic eyes, 40 mmHg in aphakic eyes and 50 mmHg in eyes that underwent combined surgery - cataract and transplant [**2**]. Subsequently, different studies showed a variable incidence of glaucoma after PK, ranging from 9 to 31% early postoperatively and from 18 to 35% in the late postoperative period [**3**,**4**]. One of the reasons for this great variation of incidence is the different manner in which glaucoma after PK is defined in various studies [**5**].

## Definition

Glaucoma is defined as a chronic progressive optic neuropathy that has characteristic morphological changes of the optic nerve and of the retinal nerve fiber layer in the absence of other ocular diseases and congenital anomalies. Starting from this classical statement, defining glaucoma after PK implies numerous difficulties: difficulty in performing a preoperative examination of the optic nerve, visual field and even of the IOP (the cornea is usually opaque); the postoperative examination can also be troublesome (high astigmatism, reduced transparency of the cornea).

Therefore, multiple studies define post-PK glaucoma as an elevation of IOP greater than 21 mmHg, independent of the optic nerve or visual field modifications. The definition obviously has scientific deficiencies, but has an important practical component [**3**,**6**].

A problem appears in the cases with preexisting glaucoma. Some studies enclose all these cases in post-KP glaucoma, meanwhile others include only the cases that require additional antiglaucomatous therapy (i.e. medications, laser or surgical treatment) to maintain IOP at adequate values (escalation of glaucoma therapy).

## Risk factors

Recognition of risk factors is important for the prevention, diagnosis, and early treatment of post-PK glaucoma.

The most significant risk factors are preexisting glaucoma, lens status (i.e. aphakia, pseudophakia) and the disease for which PK is performed [**7**].

In a study from 2014, Hemanth et al. compared the incidence of glaucoma after PK in phakic, pseudophakic, and aphakic eyes. The aphakic group had the highest risk, followed by the pseudophakic and phakic group; there was no statistically significant difference between the last two groups [**3**].

Kirkness and Ficker published one of the greatest studies on the incidence and risk factors associated with post-PK glaucoma, that included 1122 PKs, performed at Moorfields Eye Hospital, London. The incidence of post-PK glaucoma was 14%. Corneal dystrophies and keratoconus had the lowest risk of glaucoma, contrary to bullous keratopathy, anterior segment trauma, iridocorneal endothelial syndrome and corneal perforations that had an increased risk [**8**,**9**]. In another study, Kirkness and Mashegov demonstrated an increased incidence of post-PK glaucoma after corneal perforations, especially those after bacterial ulcers, due to the formation of peripheral anterior synechiae (PAS). The longer the period between the perforation and the transplant, the higher the risk of glaucoma [**10**].

## Pathogenesis

The pathophysiology of post-PK glaucoma is multifactorial, including among the causes the compression of the angle’s anatomical elements with the trabecular meshwork’s (TM) collapse, incorrect suture of the graft, postoperative inflammation and prolonged use of corticosteroids in the postoperative treatment.

Other causes are not specific to post-PK glaucoma, also appearing after other types of surgical interventions; they should be promptly recognized and treated: pupillary blockage, lens induced glaucoma, hyphaema and viscoelastic retention [**5**].

Zimmerman et al. demonstrated how the TM’s collapse, especially in aphakic eyes, is the main cause of glaucoma; they advocated that for an easy access through the anterior chamber angle to the trabecular meshwork, the trabeculum needs a posterior fixation, sustained by the ciliary body-lens complex and an anterior fixation at the level of Descemet membrane (DM). In aphakic and pseudophakic eyes the posterior support is relaxed due to lens removal. On the other side, the incision through DM in PK relaxes the anterior support, the DM being capable of displacing towards the angle [**11**].

In 1975, using a mathematical model, Olson and Kaufman pointed out the factors that contribute to the distortion of the angle and so to the reduction of the outflow: tight sutures (widen the gap between the margins of the incision in the DM), lengthy sutures (compress even more the tissues), large grafts and thick peripheral corneas [**12**].

One of the causes of late glaucoma after PK is the formation of PAS. They can appear if the anterior chamber is shallow prior to the intervention (e.g. corneal perforation) or postoperatively (e.g. wound dehiscence or iris incarceration at the graft-host junction).

Long-term use of corticosteroids, necessary for graft rejection prevention, can lead to IOP elevation. Corticosteroids that increase the IOP to a lower extent (e.g. fluorometholone) can be used, but these are less effective in rejection prophylaxis.

## Diagnosis

Measuring the IOP, optic disc and visual field evaluation are in the most occasions difficult to perform preoperatively, because of the corneal disease, which makes it impossible to establish a starting point for the postoperative period.

Changes in the corneal thickness, high astigmatism and other refractive errors, that also harden a proper evaluation, appear after PK.

In the immediate postoperative period, the diagnosis is based principally on measuring the IOP; later, to the extent possible (transparency of the cornea and ocular media), the classical evaluation is approached [**1**].

High astigmatism, graft edema, thick fluorescein meniscus and inappropriate mires (especially for grafts smaller than 7.5 mm) make Goldmann applanation tonometry (GAT) practically impossible [**1**]. In cases of irregular surface, IOP can be measured with the Mackay-Marg electronic tonometer, the pneumotonometer [**13**] or the Tono-Pen [**14**]. Comparing the iCare tonometer with the GAT, Salvetat et al. demonstrated that the values are comparable in cases of anterior and posterior lamellar keratoplasty, but are different in cases of PK, the iCare tonometer underestimating pressure compared to the GAT [**15**]. Epithelial and stromal edema lead to the under evaluation of IOP and the measurement on a scarred tissue can give false raised values. Dynamic contour tonometer was imagined for measuring IOP independent of the mechanical properties of the cornea, which theoretically makes it very useful in eyes with keratoplasty, in which these properties are profusely modified. A digital piezoelectric pressure transducer is directly coupled to the ocular surface contour and directly measures the IOP transcorneally. More studies showed that this tonometer’s measurements are closer to the true manometric measurements than the other ones are [**5**]. Comparing the dynamic contour tonometer and the GAT in cases with keratoplasty, Ceruti et al. proved that the tonometer was not influenced by thickness, curvature, and corneal astigmatism [**16**].

## Treatment

**Prophylaxis**

Preexistent glaucoma should be well controlled prior to the surgical intervention. If the IOP is difficult to control with drugs or if the control imposes maximal therapy, the IOP can decompensate after keratoplasty. Therefore, it is recommended that in these cases, the glaucoma should be controlled surgically and afterwards the transplant should be performed [**5**], because multiple studies revealed a higher incidence of graft failure if the intervention for glaucoma was performed after keratoplasty [**17**]. Other authors recommend trabeculectomy with mitomycin C (MMC) application or artificial drainage systems concomitantly with PK [**17**-**19**].

In the case of PK, certain elements of the surgical technique can lower the risk of postoperative glaucoma. A graft 0.5 mm larger than the host opening is associated with a lower frequency of glaucoma after PK [**20**,**21**]. Deep sutures allow a better coalescence of the host and graft DM [**11**] and relatively short and equal sutures between graft and host compress the tissues to a lesser extent, both elements attenuating the TM’s tendency to collapse. In case of PAS, goniosynechialysis can be performed. Viscoelastic substances should be removed as completely as possible at the end of the intervention. In case of atrophic iris or floppy-iris syndrome, iridoplasty can be performed to tension the iris and to remove it from the angle [**22**]. Graft suture should be tight, avoiding iris incarceration at the graft-host junction, as well as the dehiscence of the plague postoperatively.

A frequent instillation of corticosteroids in the immediate postoperative period controls inflammation, reducing the risk of PAS. Dosage must be carefully monitored, because in the mean and long term, corticosteroid induced elevation of pressure can appear. Also, in the early postoperative period, mydriatics can also be used to prevent pupillary blockage.

**Medical treatment**

Medical treatment represents the first line of therapy in preventing glaucoma after PK. Fortunately, many classes of drugs are at our disposal as compared to 20 years ago: beta-blockers, alpha-2 agonists, carbonic anhydrase inhibitors (CAI) and topical prostaglandin analogues, as well as systemic CAI. Miotics and adrenergic agents have historical value at the most, being no longer used. Therapy must be thought in such a manner that is efficient, but with the lowest rate of adverse effects on the graft and on the patient’s quality of life.

Beta-blockers are effective in lowering IOP and the effect installs rapidly. This group of agents can be utilized for IOP spikes in the perioperative period, but also for long-term therapy. In prolonged use, the adverse effects are punctate epithelial keratopathy and corneal anesthesia that can affect the graft’s epithelium, compromising it [**23**].

Alpha-2 agents also act promptly, are efficient and have few systemic adverse effects. Approximately one quarter of patients have allergies that lead to discontinuation of treatment.

Topical CAI are well tolerated, do not have the systemic adverse reactions of the systemic administration and can be combined with other topical antiglaucomatous drugs. However, carbonic anhydrase has a role in the pump function of the corneal endothelium. Some studies showed endothelial decompensation with corneal edema in cases of borderline endothelial function. For this reason, it is recommended to stop therapy when edema of the corneal graft appears [**5**].

Prostaglandin analogues efficacy is renowned, are administered once daily and have few systemic adverse effects. Because the effect installs relatively slow, these agents are suited for chronic forms of post-PK glaucoma. They should be avoided in PK for herpetic keratitis, because recurrence of herpetic keratitis has been reported and also the possibility of inducing cystoid macular edema should be taken into account [**24**,**25**].

Systemic CAI are very efficient, have the advantage of not having any direct toxic effect on the graft, but are difficult to utilize on the long term, 30-50% of the patients manifesting side-effects such as: paraesthesia, tinnitus, fatigue, muscular weakness, nausea, depression and cutaneous allergies. They are very useful in the treatment of sudden IOP spikes in the immediate postoperative period.

**Laser treatment**

Nd:YAG laser iridotomy can be used in pupillary blockage, but in the majority of the cases cannot be performed properly, because the patient’s peripheral cornea is not sufficiently transparent. For the same reason, neither Argon laser iridoplasty can be performed, which is useful in peripheral iris retraction and obtaining an easier access to the trabeculum.

Argon or selective laser trabeculoplasty can be used in selected cases, with transparent graft, open angle, moderate pressure (25-30 mmHg) with medical treatment at a few months after keratoplasty, that allow the application of the lens. Van Meter et al. reported IOP control with Argon laser trabeculoplasty in 10 out of 14 cases for 2 years [**26**]. Considering that the effect of Argon laser trabeculoplasty is limited in time, that it can lower IOP with a maximum of 8-10 mmHg and that in the majority of cases, the angle of PK patients has PAS and a tendency for collapse of the TM, with difficult visibility, we consider that this option of therapy should be avoided in the treatment of glaucoma after PK.

**Surgical treatment**

**Trabeculectomy**

Standard trabeculectomy has a low rate of success due to extensive subconjunctival fibrosis (after prior interventions) and the presence of PAS that numerous times are extended towards the graft-host junction and make the functioning of the fistula difficult. A study performed on 35 PK patients showed that 92% of them required medication after trabeculectomy and 53% necessitated further surgical interventions [**27**].

5-fluorouracil (5-FU) can be administered for many days postoperatively via subconjunctival injections. It has a toxic effect on the corneal epithelium and can compromise the graft. The incidence of epithelial toxicity can be as much as 50% of the cases [**28**].

Because of the corneal toxicity of 5-FU, most surgeons prefer MMC [**7**]. It is applied for 1 to 4 minutes intraoperatively and does not appear to have toxicity on the corneal epithelium. In a retrospective study on patients with glaucoma after PK, trabeculectomy with MMC had a higher success rate concerning IOP control (73%) in comparison to standard trabeculectomy (25%) for a follow-up period of 22 months [**29**]. Clarity of the graft was present in 69.2% of the cases from the lot with MMC and in 37.5% of the cases from the standard trabeculectomy lot. On the other side, the MMC group had a greater incidence of choroidal detachment, macular edema and fistula of the filtering bleb.

All measures for preventing anterior chamber collapse intra- and postoperatively should be taken. Endothelial cell loss after trabeculectomy is of 7-12% in case of iridocorneal contact and of 40-50% in case of corneolenticular contact [**30**]. This loss of endothelial cells can compromise the graft. In order to assure a relatively deep postoperative chamber, an over-tight suturing of the scleral flap and subsequent laser lysis of these sutures can be considered.

Another element that should be monitored after trabeculectomy in cases with a more prominent bleb is the dellen effect that can lead to epithelial defect and appearance of a corneal ulceration in the suture area.

**Artificial drainage devices**

From a theoretical standpoint, inserting an artificial drainage device has some advantages compared to trabeculectomy. The filtering bleb is located posteriorly, possibly avoiding perilimbal fibrosis. Overfiltration with postoperative hypotony and collapse of the anterior chamber can be avoided either by using devices with valves (i.e. Ahmed implant) or by provisory suturing of the tube (i.e. Molteno implant). The success rate in reducing IOP after PK is 60 to 80% [**31**,**32**].

Artificial drainage devices have their disadvantages. They are more expensive, require a higher surgical experience and the most important, have a relatively higher risk of graft failure than trabeculectomy [**31**-**33**].

A mechanism of graft failure in eyes with artificial drainage devices could be immunological; the tube apparently allows the access of inflammatory cells from the subconjunctival space into the anterior chamber that could contribute to graft rejection [**34**]. The most important mechanism is the mechanic one, the tube being implanted in the anterior chamber and even though at the moment of implantation it is distant from the graft’s endothelium, it can displace afterwards.

To avoid injuring the endothelium, the valve’s tube can be implanted at the level of the pars plana. The success rate in the control of IOP is similar, but there is a higher rate of retinal complications (e.g. cystoid macular edema, epiretinal membranes, retinal detachment, suprachoroidal hemorrhage) [**35**].

**Cyclodestructive procedures**

Cyclocryotherapy has been used from the 1950s in the treatment of refractory glaucoma. The success rate in the control of IOP is satisfying (60-90%), but is mainly replaced by cyclophotocoagulation, because of the great rate of serious complications [**36**,**37**].

Nd:YAG laser cyclophotocoagulation is a more predictable method, less painful and with a lower rate of complications [**38**,**39**]. The success rate in IOP control varied from 69 to 77% with a follow-up period of 2 years. However, a high rate of graft failure (23-46%) appears and also, other severe complications such as: hypotony, phthisis bulbi [**38**,**39**].

Diode laser cyclophotocoagulation with a 810 nm wavelength has some advantages: the laser is portable, easier to manipulate and as effective as the Nd:YAG laser. Okacoglu et al. showed a rate of success of 92% in IOP control at 6 months and of 72% at 12 months in patients with post-PK glaucoma. 44% of the patients had to be retreated to obtain the control of the IOP, but no graft rejection was reported in the 32 patients of this study [**40**].

It is difficult to assess which surgical treatment option - trabeculectomy with antimetabolites, implantation of an artificial drainage device or cyclophotocoagulation - is the most useful in the treatment of glaucoma after PK. As demonstrated, there are numerous studies on series of cases, generally retrospective, but there are no prospective, randomized studies that compare the treatment options.

From our point of view, the future belongs to cyclophotocoagulation. Trabeculectomy with antimetabolites, as well as artificial drainage devices interfere with the anterior chamber and any intervention at this level increases the risk of graft failure. Probably a perfected variant of the existing cyclophotocoagulation lasers that would reduce the rate of complications related to hypotony, even if the intervention was periodically repeated, would theoretically have the highest chances of success.

## Conclusions

Glaucoma is a complication of PK very difficult to treat. Identifying the risk factors allows an attentive follow-up and rapid treatment of the postoperative IOP rises. Possibly, the most important fact is that preexisting glaucoma should be rightly diagnosed and controlled before PK, medically or, if necessary, surgically.

Certain technical details can reduce the incidence of TM’s collapse and implicitly the appearance of glaucoma after PK.

Once the glaucoma has installed, the medical treatment can control the IOP in certain cases and antiglaucomatous surgical interventions also contribute to the control of IOP.

Graft failure can appear relatively frequently after surgical interventions, especially in those in which important inflammation at the level of the anterior chamber or mechanical injury of the corneal endothelium develop.

**Disclosures**

None of the authors has any financial or proprietary interests to disclose.
